# Cognitive Behavioral Therapy in Endometriosis, Psychological Based Intervention: A Systematic Review

**DOI:** 10.1055/s-0042-1742406

**Published:** 2022-05-16

**Authors:** Lilian Donatti, Helena Malvezzi, Bruna Cestari de Azevedo, Edmund Chada Baracat, Sergio Podgaec

**Affiliations:** 1 Departamento de Ginecologia, Hospital das Clínicas, Faculdade de Medicina, Universidade de São Paulo, São Paulo, SP, Brazil; 2 Hospital Israelita Albert Einstein, São Paulo, SP, Brazil

**Keywords:** endometriosis, cognitive behavioral therapy, psychological intervention, chronic pelvic pain, quality of life, systematic reviews, endometriose, terapia cognitivo-comportamental, intervenção psicológica, dor pélvica crônica, qualidade de vida, revisões sistemáticas

## Abstract

**Introduction** Endometriosis is an inflammatory disease that affects women of reproductive age, causing pain and the possibility of infertility. Endometriosis was associated to low life quality and research shows the impact of endometriosis in several areas of life, justifying how these patients are more likely to develop depression, anxiety, and stress.

**Objective** The aim of the present systematic review was to explore the field of psychology in endometriosis, identifying studies that used the cognitive behavioral therapy technique as a treatment for endometriosis and chronic pelvic pain.

**Methods** The keywords used were *Endometriosis* and *Behavioral* Therapy; *Behavioral Disciplines and Activities* ; *Cognitive Behavioral Therapy* ; *Mental Health* ; *Psychological Techniques* ; *Psychology* ; *Psychotherapy* ; *Mental Health Services* ; and the search was performed in the following databases: PubMed/Medline, Scielo, Lilacs, and Capes. The study followed the PRISMA guidelines and all studies whose intervention strategy used was related to cognitive-behavioral therapy were considered.

**Results** Of the 129 articles found, only 5 were selected, and it was possible to identify that the psychological intervention whose approach brought cognitive-behavioral therapy techniques promoted a decrease in the sensation of pain, improvements in the scores of depression and stress, and significant changes in aspects of quality of life such as vitality, physical and social functioning, emotional well-being, control, and autonomy.

**Conclusion** Cognitive-behavioral therapy can be very promising to take care of the emotional side of those who have endometriosis However, the present systematic review highlights the need to develop more structured studies with consistent, clear and replicable methods to reach a psychological intervention protocol for patients who live with this gynecological-physical-emotional condition.

## Resumo

**Introdução** A endometriose é uma doença inflamatória que afeta mulheres em idade reprodutiva, causando dor e possibilidade de infertilidade. A endometriose foi associada a baixa qualidade de vida e pesquisas mostram o impacto da endometriose em diversas áreas da vida, justificando como tais pacientes têm maior probabilidade de desenvolver depressão, ansiedade e estresse.

**Objetivo** O objetivo da presente revisão sistemática foi explorar o campo da psicologia na endometriose, identificando estudos que usaram a técnica da terapia cognitiva comportamental como tratamento da endometriose e da dor pélvica crônica.

**Métodos** As palavras chaves utilizadas foram *Endometriose* AND *Terapia* *comportamental* ; *Disciplinas e atividades comportamentais* ; *Terapia cognitiva comportamental* ; *Saúde mental* ; *Técnicas psicológicas* ; *Psicologia* ; *Psicoterapia* ; *Serviços de saúde mental* , e a busca foi realizada nos bancos de dados PubMed / Medline, SCIELO, LILACS e CAPES. O estudo seguiu as diretrizes dos Principais Itens para Relatar Revisões Sistemáticas e Metanálises (PRISMA, na sigla em inglês) e foram considerados todos os estudos cuja estratégia de intervenção utilizada estava relacionada à terapia cognitivo-comportamental.

**Resultados** Dos 129 artigos encontrados, somente 5 foram selecionados, e foi possível identificar que a intervenção psicológica cuja abordagem trouxe técnicas da terapia cognitivo-comportamental promoveu diminuição na sensação de dor, melhora nos escores de depressão e estresse e mudanças significativas em aspectos da qualidade de vida como vitalidade, funcionalidade física e social, bem-estar emocional, controle e autonomia.

**Conclusão** A terapia cognitivo-comportamental pode ser muito promissora para o tratamento psicológico/emocional de quem tem endometriose. No entanto, a presente revisão sistemática destaca a necessidade de desenvolver estudos mais estruturados com métodos consistentes, claros e replicáveis para se chegar a um protocolo de intervenção psicológica para pacientes que convivem com esse quadro ginecológico-físico-emocional.

## Introduction

Endometriosis is an inflammatory gynecological disease that affects one in ten women of reproductive age and is characterized by the presence of endometrial tissue outside the uterus, causing inflammation, pain, and infertility. 1

Endometriosis is considered an enigmatic disease that is difficult to diagnose, taking ∼ 7 to 10 years to be identified. 2 However, with scientific and technological advances, there has been an improvement in medical diagnosis and in the dissemination of knowledge and understanding of the disease.

The main symptoms of endometriosis are pelvic pain and infertility. 3 4 Currently, if a patient is treated by an endometriosis specialist practitioner and imaging exams (transvaginal ultrasound with bowel preparation or magnetic resonance imaging [MRI]) are made by specialist radiologists, 5 6 the diagnosis is accurate and faster. The biggest challenge today is no longer the effectiveness of medical diagnosis or treatment, but the access of the general population to this scenario.

Treatment protocols are well-established for patients with endometriosis, including hormones to block menstruation, analgesics and anti-inflammatories to control pain; and surgical procedures for the excisions of lesions, usually done by videolaparoscopy. 4 7

The path to medical diagnosis and treatment may be well traced, but the main issue is how each woman will deal with the disease. There is still a great challenge to improve the quality of life of these patients, who end up having numerous social, professional, physical, financial, emotional, and sexual life consequences. 8

There are studies that link endometriosis feature to low quality of life and the impact caused by the disease. 8 9 Furthermore, it is known that women with endometriosis are more likely to develop depression, anxiety, and stress compared with those without the disease. 8 10

Several scientific articles indicate the effectiveness of using techniques to relieve this clinical condition and improve the quality of life, such as physical therapy, 9 nutritional guidance, 11 physical exercise, 12 Yoga practice, 13 progressive muscle relaxation, 14 acupuncture, 15 16 mindfulness, 17 18 and psychotherapy, 9 16 19 which demonstrate the need for multidisciplinary treatment of endometriosis. Cognitive behavioral therapy (CBT), one of the psychological treatments used for endometriosis patients, is based on a conceptualization or understanding of each patient (their specific beliefs and behavior pattern), where, in several ways, the psychotherapist seeks to produce cognitive changes on the thinking and belief system of the patient to produce lasting emotional and behavioral changes. 20

However, there is still not enough data in each of these categories or validated protocols to be widely recommended. 21

Thus, the purpose of the present systematic review is to explore the field of psychology, and to identify studies that use CBT technique in individual or group interventions in the treatment of women with endometriosis and chronic pelvic pain.

## Methods

### Literature Search

The present study was performed at Department of Obstetrics and Gynecology of the Hospital das Clinicas of the Faculdade de Medicina of the Universidade de São Paulo, following the PRISMA 22 statement for systematic reviews. The keywords used were “Endometriosis” and “Behavioral Therapy”; “Behavioral Disciplines and Activities”; “Cognitive Behavioral Therapy”; “Mental Health”; “Psychological Techniques”; “Psychology”; “Psychotherapy”; “Mental Health Services”; the research was performed in PubMed/Medline, Scielo, Lilacs and Capes databases from June 20, 2019, until July 30,2020 ( Table 1 ).

**Table 1:** Search method for PubMed/Medline, SciELO, Lilacs, and Capes

Scientific databases	Search period	Search terms
PubMed/Medline Scielo Lilacs Capes	June 20, 2019, until July 30, 2020	*Endometriosis* *Behavior* *Therapy* *Behavioral* *Disciplines* *and* *Activities* *Cognitive* *Behavioral* *Therapy* *Mental* *Health* *Psychological* *Techniques* *Psychology* , *Psychotherapy* *Mental Health Services*

### Eligibility Criteria

Only studies whose intervention strategy was to approach patients with endometriosis with CBT techniques were contemplated and no data limits were imposed. Studies that presented other intervention strategies rather than CBT were not considered. Manuscripts that present data on well-structured interventions in individual or group sessions with the use of psychological interventions based on CBT techniques in patients were selected and there were no restrictions based on the topics worked during the interventions, as long as the CBT technique was applied. The reasons for the exclusion of article were mixed techniques; guidelines for painful; other language; old studies; no intervention; review articles; animal studies; focus pain and adolescence.

### Data Extraction and Synthesis

Two reviewers, individually, performed the eligibility assessment of articles in an unblinded standardized manner, and any divergence on the inclusion of studies was solved by consensus. A systematic review was performed by analyzing the number of patients, the type of study, the results, the conclusions of each study, and which instrument and intervention were evaluated.

## Results

**Fig. 1:**
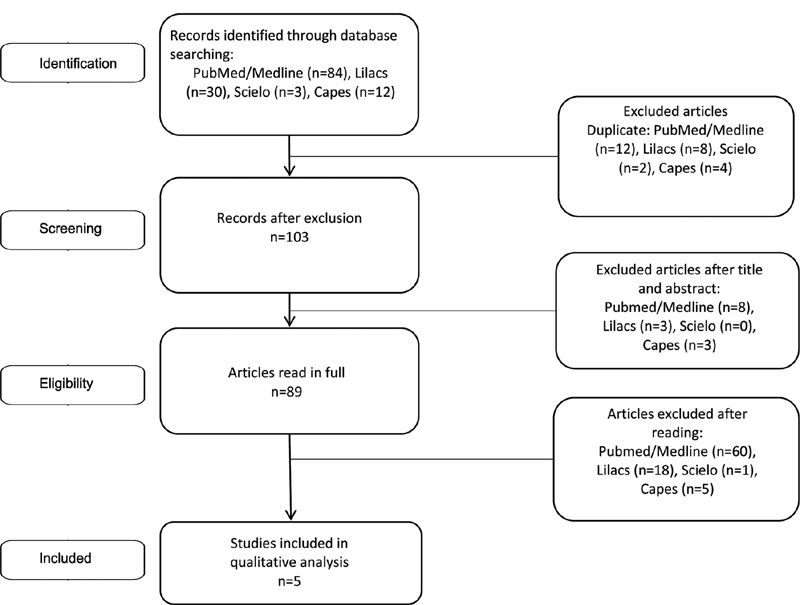
Flow diagram of the studies included in the systematic review, after searching for the focal points *Endometriosis* ; *Behavioral Therapy* ; *Behavioral Disciplines and Activities* ; *Cognitive Behavioral Therapy* ; *Mental Health* ; *Psychological Techniques* ; *Psychology* ; *Psychotherapy* ; *Mental Health Services* and excluding duplicates, reviews, and discordant themes.

A total of 129 articles were retrieved from database searching: 84 from PubMed/Medline, 30 from Lilacs, 3 from Scielo, and 12 from Capes. After excluding duplicate articles, the final list produced 103 articles. Titles and abstracts of all studies identified by the search strategy were examined and 89 relevant papers were read in full. At the end, five articles that met the investigation criteria of the present systematic review were selected ( Fig. 1 ). A meta-analysis could not be performed because it is necessary a minimum of two studies with information on the outcome of interest and effect size in the same direction, or a minimum of three studies, regardless of the direction of effect estimation, and the five articles presented heterogeneous results.

Five studies that presented intervention strategies within the psychological approach of CBT were selected; each article had specific characteristics and described important results in different contexts ( Tables 2 and 3 ).

**Table 2:** Details of included studies

Source	N treatment	N control	Age (years old)	Study Design	Inclusion criteria	Exclusion criteria	Instrument used	Other intervention strategy applied in addition to psychology	Intervention period and organization	JOURNAL	Limitation
Lorençatto et al. (2007) 9 Brazil	64	64	median 34,7	Randomized clinical trial	Surgical diagnosis of endometriosis, symptons of chronic pelvic pain at the beginning of the group evaluation, having participated in at least seven meetings, having completely completed the clinical and psychological assessments and having completed the daily pain record	Those who had already participated in other psicological intervation	Sociodemographic questionnaire, clinical condition of endometriosis and pain symptom; Beck Depression Inventory (BDI); Visual Analogue Scale (VAS); daily pain record	1 hour of physiotherapy before psychology sessions	10 sessions in groups of 12 patients	Rev Assoc Med Bras	No protocol application specification session by session
Meissner et al. (2016) 16 Germany	35	32	18 to 40	Randomized clinical trial	Adult women aged between 18 and 40 years old with a history of histologically verified endometriosis and chronic pelvic pain	Hormonal treatment during the month prior to enrollment, use of drugs or alcohol, pregnancy, insufficient knowledge of the German language and contraindications for MRI (as a result of the primary outcome)	Magnetic resonance imaging; numerical scale from “0” (“without pain”) to “10” (“worst possible pain”); quality of life (scores of the physical and psychological sum of the German version 12 Item: Short-Form Health Survey); functional well-being, depression, and anxiety and measurement for acute pain	Hypnotherapy and acupuncture	Individual sessions of 30 to 60 minutes. 3 months application and follow-up after 6 and 24 months	Obstetrics & gynecology	No protocol application specification session by session
Kold et al. (2012) 17 Denmark	10	0	14 to 37	Qualitative study. Prospective, observational, pilot study	Endometriosis diagnosis and severe pain	not mentioned	SF36 and EHP-30	No	10 sessions (5 in group and 5 individually: total of 15 hours) and 6 and 12 months of follow-up	Nordic psychology	No control group
Hansen et al. (2016) 18 Denamark	10	0		Longitudinal study	Patients who had participated in the 2012 intervention and had committed themselves to applying the techniques at home in their daily lives	Patients who failed to commit to practicing mental techniques at home	EHP-30 and SF-36	No	10 weeks, follow-up up after 6 years	Nordic psychology	No control group
Friggi Sebe Petrelluzzi et al. (2012) 19 Brazil	26	0	median 32,2	Observational, prospective	Endometriosis diagnosis by visual identification during laparoscopy or laparotomy plus biopsy; and with chronic pelvic pain	Patients who abandoned treatment for some reason or who did not have a frequency of at least 75% during sessions	Visual Analog Pain Scale (VAS), Perceived Stress Questionnaire (PSQ), SF36, Cortisol levels in saliva.	During the 1st hour: physical therapy, lumbar repositioning, body awareness, breathing exercises (focusing on rhythm, frequency, depth, volume of air inhalation and respiratory rehabilitation), stretching, exercises aiming the pelvic region, perineal muscle strengthtening, massage, self-massage, transcutaneous electrical nerve stimulation, relaxation and instructions related to activities of daily life	10 sessions, once a week (2h30m)	Journal of Psychosomatic Obstetrics & Gynecology	No control group

**Table 3:** Intervention results

Results	CBT techniques	Source
Decreased pain scores	Cognitive and behavioral techniques/psychoeducation	Lorençatto et al. (2007) 9
	Mindfulness/coping/psychoeducation	Kold et al. (2012) 17
	Mindfulness/coping/cognitive and behavioral techniques	Meissner et al. (2016) 16
Decreased depression score	Cognitive and behavioral techniques/psychoeducation	Lorençatto et al. (2007) 9
Reduction in the perception of stress	Psychoeducation/coping	Friggi Sebe Petrelluzzi et al. (2012) 19
Normalization of cortisol levels	Psychoeducation/coping	Friggi Sebe Petrelluzzi et al. (2012) 19
Increased vitality in everyday life	Psychoeducation/coping	Friggi Sebe Petrelluzzi et al. (2012) 19
Increased day-to-day functionality	Psychoeducation/coping	Friggi Sebe Petrelluzzi et al. (2012) 19
	Mindfulness/psychoeducation	Kold et al. (2012) 17
Increased sense of well-being	Mindfulness/coping/psychoeducation	Kold et al. (2012) 17
Improvement in quality of life	Mindfulness	Hansen et al. (2016) 18
	Mindfulness/coping/cognitive and behavioral techniques	Meissner et al. (2016) 16

Group intervention and physical therapy was the CBT used in the study by Lorençatto et al., 9 a randomized clinical trial. In the psychological field, CBT, recreational, and occupational activities were applied, and psychoeducational material was used as additional reading directed to the topics of the session. The points covered were self-knowledge (correlation between body and mind, acceptance, meaning, and coping with illness, pain, and stress), theory and management of symptoms, endometriosis (current concepts and available treatments), sexuality, family and social affective relationships, and problem-solving strategies. After the end of the intervention process, a decrease in pain and depression scores was observed among patients.

Friggi Sebe Petrelluzzi et al. 19 performed CBT as coping strategies and used psychoeducational material in a prospective and observational study. The topics covered were endometriosis, pain, stress, family, social relationships, and sexuality. In addition to the psychological strategies, during the first hour of the session, physical interventions were applied: lumbar repositioning, body awareness, breathing (focusing on rhythm, frequency, depth, volume of inhaled air, and respiratory rehabilitation), stretching, pelvic exercises, strengthening of the perineal muscle, massage, self-massage, transcutaneous electrical nerve stimulation, relaxation, and instructions focused on daily living activities. The results obtained showed a reduction in the stress perception, normalization of cortisol levels, and increased daily functionality and vitality.

Furthermore, Kold et al., 17 in a prospective and observational study, applied the mindfulness technique (body scanner, sensory training, breathing techniques, music, and biofeedback), considered to be a third wave of CBT (new generation), psychoeducation, counseling, and support. The topics covered during sessions were grieving processes, emotions associated with adaptation to chronic pain, stress and pain, work issues, healthy habits, including diet and exercise, social networking in a challenging situation and mind-body interaction. The results indicated significant and lasting effects on reducing the pain level and an improvement well-being and daily functionality.

The longitudinal study by Hansen et al. 18 also applied the mindfulness technique on a continuous daily basis. The focus topics were pain, coping, fatigue, sleep quality, work, and relationship and family issues. A lasting improvement in the quality life was observed.

Meissner et al., 16 in a randomized clinical trial, applied a body and mindset: psychotherapy using CBT techniques including mindfulness and problem solving. In addition to psychological strategies, hypnotherapy, and acupuncture techniques such as moxibustion (a type of thermal acupuncture, made by combustion of herbs), and suction cups were also used. The topics covered were pain and quality of life. The researchers found an overall decrease in pain, pelvic pain, dyschezia, and an improvement in the quality of life.

Even in the face of a diversity of techniques and contexts, when compiling data ( Tables 4 and 5 ), it was possible to observe gain in the results where there was a psychological intervention whose approach applied CBT techniques. As a result, patients had a decreased in pain sensation, improvement in depression and stress scores, and significant changes in aspects of quality of life, such as vitality, physical and social functioning, emotional well-being, control, and autonomy.

**Table 4:** What improved in depression, stress, and pain

Source	What improved			
	Depression	Stress	Pain	
			Pelvic pain	Dyschezia
Lorençatto et al. (2007) 9	22.8 ± 10.2 versus 17.0 ± 10.2 ( *p* < 0.0001).	X	4.2 ± 3.3 versus 6.6 ± 2.4 ( *p* = 0.0002) general	X
Meissner et al. (2016) 16	X	X	(-1.4. 95%CI: 2.7–- 0.1; *p* = 0.036) pelvic	(-3 .5. 95% CI: - 5.8–-1.3; *p* =.003)
Kold et al. (2012) 17	X	X	52.53 ± 12.52 versus 33.18 ± 15.46; *p* > 0.005	X
Hansen et al. (2016) 18	X	X	- 10.83 versus - 18.1; *p* = 0.583	X
Friggi Sebe Petrelluzzi et al. (2012) 19	X	Acknowledge stress: 0.62 ± 0.02 versus 0.56 ± 0.02; *p* < 0.05	X	X

Abbreviation: CI, confidence interval.

Initial (before intervention) versus Final (after intervention)

**Table 5:** What improved in life quality

Source	What improved						
	Vitality	Physical functioning domains	Control and incapacity	Emotional well-being	Social support	Corporal pain	Physical and emotional role
Lorençatto et al. (2007) 9	X	X	X	X	X	X	X
Meissner et al. (2016) 16	X	3.8; 95%CI: 0.5–7.1; *p* = 0.026	X	5.9, 95%CI: 0.6–11.3; *p* = 0.031	X	- 2.5; 95%CI: - 3.5– -1.4; *p* < .001	X
Kold et al. (2012) 17	X	69.34 ± 14.62 versus 77.00 ± 18.14; *p* > 0.05	65.28 ± 18.98 versus 37.50 ± 10.58; p > 0.005	52.08 ± 16.23 versus 34.17 ± 15.06; p > 0.008	52.50 ± 25.89 versus 31.25 ± 15.59; p>0.005	23.00 ± 27.51 versus 61.00 ± 8.76; *p* > 0002;	23.33 ± 35.31 versus 76.67 ± 35.31; *p* > 0.05;
Hansen et al. (2016) 18	- 23.28 versus -10.28; *p* = 0.404	- 12.07 versus -20.01; *p* = 0.587	- 12,24 versus -19.74; *p* = 0.609	- 18.08 versus -6.08; *p* = 0.290	−26.00 versus (-1.50) *p* = 0.032	- 22.82 versus - 20.22; *p* = 0.894	- 65.28 versus -11.95; *p* = 0.153
Friggi Sebe Petrelluzzi et al. (2012) 19	30 ± 3.9 versus 39 ± 4.6; *p* < 0.05	26 ± 6.5 versus 38 ± 7.5; *p* < 0.05	X	X	X	X	X

Abbreviation: CI, confidence interval.

Initial (before intervention) versus Final (after intervention).

## Discussion

The present systematic review assessed studies that used CBT techniques for problem solving, as the concept of coping, mindfulness, relaxation, psychoeducation, and cognitive techniques for the treatment of endometriosis patients. Cognitive behavioral therapy has become a worldwide psychotherapy widely performed and already validated for the management of the treatment of several diseases, such as depression, generalized anxiety disorder, panic disorder, social phobia, obsessive-compulsive disorder, body dysphoric disorder, personality disorders, chronic pain, migraine, and somatoform disorders, among others. 20

The increase in the use of CBT for psychology conducts is due to its contribution to the development of evidence-based psychological treatment 22 23 and, in the same way, its use is designed in the management of endometriosis.

Despite this understanding, what was observed in the present systematic review is that there are still few studies on endometriosis in the psychological field that use CBT. Vernon et al. 24 explain that CBT has become the dominant generic term used to describe a wide range of counseling and psychotherapy approaches that represent three distinct, yet overlapping, therapeutic forms: behavioral therapy, cognitive therapies, and attention and acceptance therapies.

Lorençatto et al., 9 used a group of CBT techniques in the intervention group, whereas the control received no treatment. Despite the positive points of that study, there was no protocol specification detailed session by session, which makes it difficult to be replicated. However, experience exchange can be beneficial in group dynamics as it gives rise to the idea of standardization and belonging; in other words, what a person lives and feels is also common to other people, allowing intellectual flexibility of sensations and behavior.

The concept of coping is also important, and its theories contribute to the strategy process of identifying ways to undergo stressful, crisis or unexpected situations. In this particularity, each individual develops a certain emotional pattern of conduct throughout their life, which will guide them to solve the problems that emerge. 25 Interesting studies in the field of psychology have been developed using this concept. 8 26

Friggi Sebe Petreluzzi et al. 19 used CBT and coping techniques as a strategy. However, in addition to psychoeducation, it was not clear which other CBT techniques were used to address the topics and to work on symptoms, feelings, and thoughts. Without the control group, it is also not possible to expand and ensure conclusions.

Lucena-Santos et al. 27 pointed out that the mindfulness technique, originated from the so-called “Third Generation Therapies” in CBT, comes with a proposal to better deal with internal content, instead of fighting to change uncomfortable thoughts and feelings. The new approaches focus on cultivating an attitude of acceptance without judgment in relation to all human experiences in order to increase psychological well-being.

Williams et al. 28 emphasize that, with constant practice, mindfulness can create long-term changes, including changes in mood and in levels of happiness and well-being. Scientific studies show that mindfulness not only prevents depression, but it also positively affects brain patterns that are responsible for anxiety and day-to-day stress, making it easier for this condition to dissolve once installed. Furthermore, people who regularly practice mindfulness tend to spend less time in medical appointments and hospitals. In addition, memory, creativity, and reactions improve at the same time that pain perception decreases in those who practice mindfulness.

The mindfulness technique for coping and pain was the base of CBT therapy in three studies. 16 17 18 The mindfulness technique applied in the studies by Kold et al. 17 and by Hansen et al. 18 indicated significant, positive, and lasting effects on pain levels, well-being, and functionality on a daily basis. Even more, after 6 years, these improvements were still present, thus observing a lasting change when mindfulness was employed. Both studies had no control group, which limits the proof of the results in a more comprehensive and safe way.

Meissner et al. 16 focused on the connection of body and mind to treat aspects of pain and quality of life. Despite the important results, this study, as the one by Lorençatto et al., 9 did not present the protocol specification session by session, exposing how the techniques were used throughout the meetings, making it difficult to replicate the method.

The compilation of data from the five studies selected in the present systematic review shows positive and lasting results when CBT techniques, such as psychoeducation, coping strategies, problem solving, and mindfulness in patients with endometriosis and chronic pelvic pain, are employed. These results serve as a guide for psychologists and health professionals who work with patients with this condition, showing the importance of caring for and intervening in this disease in addition to its physical aspect. We could hypothesize that, taking care of the emotional aspects of endometriosis can be a catalyst for achieving the general well-being of the patient. On the opposite side, this scenario of five studies demonstrates the lack of a pattern in the psychological treatment of endometriosis.

Although these studies have demonstrated the use of CBT technique as an important improvement in endometriosis symptoms, many limitations have been identified: the absence of a control group, no detailed description of the techniques applied, and no comparison with other approaches in psychology to ensure that CBT brought the best result. This is an important gap in the field of psychological treatment of endometriosis.

## Conclusion

The use of CBT techniques such as psychoeducation, mindfulness, and coping strategies in psychological intervention programs for patients with endometriosis and chronic pelvic pain provided an improvement in pain and depression scores, normalization of cortisol levels, which helped in a reduced perception of stress, improved the sense of vitality and functionality in daily life, increased the sense of well-being, and improved the quality of life index. Such results from these studies point to a promising path in the use of CBT strategies and techniques in the treatment of physical and psychological symptoms in women with endometriosis and chronic pelvic pain. The present systematic review highlights the need to develop more structured studies with consistent, clear, and replicable methods so that it is possible to arrive at a psychological intervention protocol for patients living with this gynecological-physical-emotional condition.
